# Navigating uncharted territory in surgical innovation: systematic review of non-standard metabolic bariatric surgery procedures

**DOI:** 10.1093/bjs/znag047

**Published:** 2026-04-16

**Authors:** Francesco S Papadia, Ottavio De Cian, Nicola Di Lorenzo, Ricardo V Cohen

**Affiliations:** Department of Surgical Sciences and Integrated Diagnostics (DISC), School of Medical and Pharmaceutical Sciences, University of Genoa, Genoa, Italy; Surgical Department, United Lincolnshire Teaching Hospitals NHS Trust, Lincoln, UK; Department of Surgery, Pietro Valdoni Institute, Sapienza University of Rome, Rome, Italy; Centre for the Treatment of Obesity and Diabetes, Hospital Alemão Oswaldo Cruz, São Paulo, Brazil

## Abstract

**Background:**

Surgical innovation often outpaces robust evaluation. This study used metabolic bariatric surgery (MBS), a field of high-intensity innovation, as a case study to evaluate reporting standards and ethical oversight for experimental procedures within surgery.

**Methods:**

A systematic review was conducted in accordance with PRISMA 2020 guidelines. PubMed/MEDLINE, the Web of Science, Scopus, the Cochrane Library, and Embase were searched from January 2000 to December 2024 using terms related to MBS, surgical innovation, and non-standard intestinal bypass procedures. Original studies reporting first-in-human or early clinical series of non-standard primary MBS involving significant intestinal modification were included and reviews, editorials, conference abstracts, revisional surgery, purely restrictive procedures, and device-based interventions were excluded. Two reviewers independently screened studies, extracted data on publication timing, patient numbers, follow-up, ethical approval, and trial registration, and assessed risk of bias using the Newcastle–Ottawa scale (NOS), the Joanna Briggs Institute critical appraisal checklist, and the ROBINS-I tool.

**Results:**

From 57 included studies (10 754 patients), the median time from first human operation to publication was 5 (interquartile range 3–8) years. The median initial cohort size was 39 (range 1–1074) patients. Institutional Review Board or ethics committee approval was reported in 47 of 57 studies (82%), while prospective clinical trial registration was documented in only 6 of 57 studies (11%). Methodological quality was low (mean NOS score of 5.0 out of 9), with 56 of 57 studies judged at high risk of bias. Out of 57 studies, only 15 (26%) reported outcomes at ≥3 years and only 8 (14%) reported outcomes at ≥5 years.

**Conclusion:**

This MBS case study reveals delayed publication and a widespread lack of prospective trial registration, exposing over 10 000 patients to experimental procedures outside a transparent research framework. These findings highlight a systemic failure in surgical innovation governance and underscore an urgent need for a cultural shift towards mandatory, prospective oversight frameworks to ensure patient safety and credible evidence generation.

## Teaser

Investigating a cohort of 57 innovative intestinal-based metabolic procedures, this review found delayed publication and a near-total lack of prospective trial registration, exposing patients to techniques without transparent evaluation. This model, characterized by low-quality evidence and high risk of bias, highlights a critical governance failure. The findings mandate a structural reform towards prospective, registered oversight for all surgical innovation.

## Introduction

Surgical innovation is the cornerstone of progress, having given rise to transformative advances such as minimally invasive surgery, solid organ transplantation, and robotic-assisted procedures. This drive to improve patient outcomes is a defining characteristic of the surgical profession. However, this virtuous cycle of innovation carries an inherent risk: the pace of technical advancement often outstrips the development of robust mechanisms for oversight, evaluation, and evidence generation^1^. This disconnect can lead to the premature adoption of techniques, under-reporting of complications, and significant ethical concerns, a pattern observed historically in fields from general surgery to surgical oncology^2^.

The field of metabolic bariatric surgery (MBS) provides a contemporary, potent, and highly relevant example of this enduring challenge within surgery. MBS is a highly effective treatment for severe obesity and related co-morbidities^3,4^, with rapid evolution over the past two decades. The pursuit of improved efficacy, reduced complications, and expanded indications has stimulated continuous innovation, leading to numerous technical variations and novel procedures^5,6^. This high rate of innovation, coupled with a large and often vulnerable patient population, makes MBS an ideal ‘stress test’ for examining the systemic processes that govern how new surgical procedures are developed, reported, and disseminated. The systemic tensions between innovation, evidence, and ethics observed in MBS are not unique—these tensions echo challenges seen in transplantation, robotic, and endovascular surgery.

History within MBS provides a cautionary tale for all surgeons; procedures like the jejunoileal bypass (JIB) were initially promising but ultimately abandoned due to severe long-term complications, including liver failure and metabolic deficiencies^7^. This pattern is not merely a historical relic, with the laparoscopic adjustable gastric band (LAGB) serving as a more recent, costly example. Initially embraced for technical simplicity and short-term safety, long-term population-level data eventually revealed profound failure rates of 18.5%, requiring reoperation, with expenditures for revisions increasing from 16% to 77% of total device-related spending over just 7 years^8^. The LAGB experience demonstrates that even a less invasive, widely adopted innovation can lead to widespread patient harm and significant financial burden on healthcare systems when long-term efficacy and safety are not adequately established before widespread adoption.

The proliferation of non-standard MBS procedures—those not yet endorsed by major international guidelines and involving substantial anatomical modifications—presents a modern iteration of this classic dilemma. The ethical introduction of experimental techniques demands adherence to principles of oversight, evidence generation, and patient safety. Frameworks for responsible surgical innovation, such as the IDEAL (Idea, Development, Exploration, Assessment, Long-term study) recommendations, have been developed^9^. IDEAL provides a structured, staged pathway for evaluation, emphasizing prospective registration, transparent reporting, and methodical progression from first-in-human cases to large-scale assessment.

This systematic review uses a defined cohort of non-standard intestinal-based MBS procedures as a case study to assess the critical question of surgical innovation aligning with established standards for responsible research having implications for all surgical disciplines. The aim of this systematic review was first to quantify the reporting patterns and ethical oversight of non-standard intestinal-based MBS procedures to assess the adherence to principles of responsible innovation, using the IDEAL framework as a benchmark. Another aim was to evaluate the implications for patient safety, professional credibility, and to propose structural reforms applicable across surgical specialties.

## Methods

This review followed the PRISMA 2020 statement^10^ and was prospectively registered in PROSPERO (CRD420250641346). Eligible studies described first-in-human or early clinical series not endorsed by the International Federation for the Surgery and Other Therapies for Obesity (IFSO) guidelines.

### Eligibility criteria

This review focused specifically on non-standard primary MBS procedures, defined as those involving significant, permanent anatomical modification of the intestinal tract that were not included in contemporary IFSO guidelines at the time of introduction. This cohort was selected as a clear, homogeneous case study of high-impact procedural innovation with distinct metabolic and nutritional long-term risk profiles, including the risk of protein-calorie malnutrition.

The inclusion criteria were as follows: original research articles (prospective or retrospective studies, case series) reporting on human subjects undergoing these operations as primary procedures, published in the English language between 1 January 2000 and 31 December 2024.
The exclusion criteria were as follows: reviews, editorials, conference abstracts, revisional surgery, procedures without intestinal alteration (for example adjustable gastric banding), and device-based interventions (for example gastric stimulation). The exclusion of devices was intentional to maintain a focus on pure procedural innovation, which often follows a less defined regulatory pathway than device-based therapies.

Procedures were classified as non-standard if not included in contemporary IFSO guidelines at the time of introduction and involved novel intestinal bypass lengths, anastomotic configurations, or interposition techniques. A priori, this included procedures such as transit bipartition (and variants like single-anastomosis sleeve ileal (SASI) bypass), ileal interposition, novel JIB variants, and duodenojejunal bypasses with significant intestinal modification. A detailed list of the procedure types identified in this review is presented in the *Supplementary material*.

### Search strategy

A comprehensive systematic search was conducted across five electronic databases: PubMed/MEDLINE, the Web of Science, Scopus, the Cochrane Library, and Embase. The search strategy combined controlled vocabulary (for example Medical Subject Heading (MeSH) terms) and keywords related to three core concepts: bariatric/metabolic surgery (for example ‘bariatric surgery’ (MeSH), ‘metabolic surgery’); innovation (for example ‘novel,’ ‘innovative,’ ‘non-standard,’ ‘new technique’); and intestinal anatomy (for example ‘intestines/surgery’ (MeSH), ‘transit bipartition,’ ‘ileal interposition’). The full search strategy for all databases is documented in the PROSPERO protocol (CRD420250641346). The detailed PubMed strategy is presented in the *Supplementary material*. Reference lists of included articles were manually screened for additional relevant studies.

### Study selection and data extraction

**Fig. 1:**
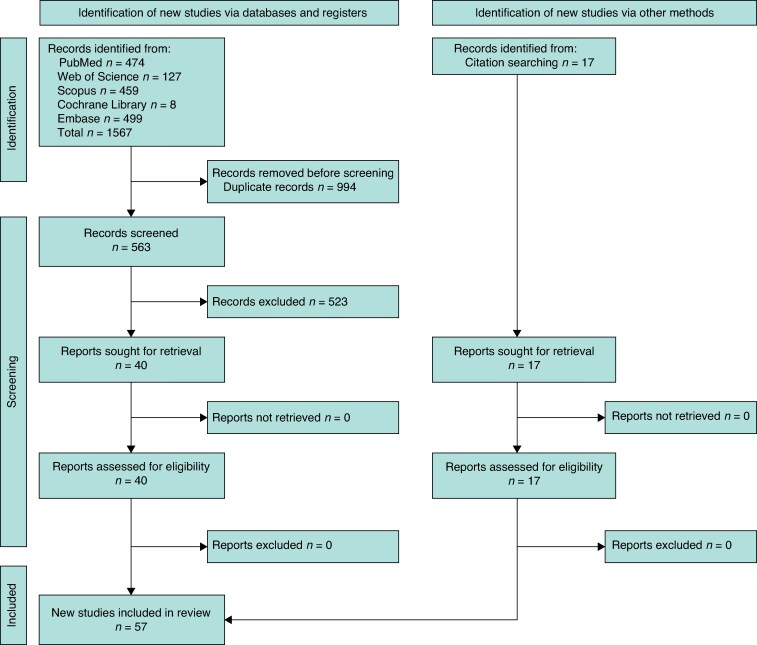
PRISMA flow diagram of study selection for this systematic review Note: An additional 21 follow-up reports were used for data extraction; these reports contributed to cumulative patient numbers and extended follow-up but are not counted as independent studies.

Two independent reviewers (F.S.P. and O.D.C.) screened titles and abstracts, followed by a full-text assessment of potentially eligible studies. Disagreements were resolved through consensus. Data were extracted using a piloted, standardized form. Key extracted variables included: study identifiers and procedure name; year of the first reported human operation and year of first publication; number of patients in the initial report and cumulative number across all identified publications for that procedure; number of performing centres; follow-up duration; reporting of Institutional Review Board (IRB) or ethics committee approval; and registration in a clinical trials database (ClinicalTrials.gov, the WHO International Clinical Trials Registry Platform (ICTRP), the European Union Clinical Trials Register (EU-CTR), the Australian and New Zealand Clinical Trial Registry (ANZCTR), or ISRCTN – The UK’s Clinical Study Registry) at the time of patient enrolment. The study selection process followed PRISMA 2020 guidelines and is summarized in *Fig. 1*. Original screening logs detailing categorical exclusion counts at the title/abstract and full-text stages were not retained after the review was completed. The flow diagram reports the total numbers of records excluded at each stage, with full-text exclusions confirmed to be based solely on the predefined exclusion criteria.

### Quality assessment

The methodological quality and risk of bias of included studies were assessed using three complementary tools: the Newcastle–Ottawa scale (NOS) for cohort studies; the Joanna Briggs Institute (JBI) critical appraisal checklist for case series; and the ROBINS-I tool.

Two reviewers independently performed the assessments, with disagreements resolved by consensus.

### Data synthesis

Due to significant clinical and methodological heterogeneity among the included procedures and studies, a meta-analysis was not deemed appropriate or feasible. Data were synthesized descriptively. Categorical variables are reported as *n* (%) and continuous variables, which were non-normally distributed, are reported as median (interquartile range (i.q.r.) and/or range).

## Results

### Study characteristics and methodological quality

The systematic search (see *Fig. 1* for the PRISMA flow diagram) identified 57 studies^11–67^, describing a diverse array of innovative procedures, which were categorized into five main groups for descriptive purposes: sleeve gastrectomy variants with small bowel resection; modifications of single-anastomosis duodenoileal (SADI) bypass; transit bipartition procedures (for example SASI bypass); modifications of JIB; and ileal interposition techniques. The key characteristics of the included studies, including procedure names, publication timelines, cohort sizes, and oversight reporting, are summarized in *Table 1*. The detailed, study-by-study quality assessments using the NOS, the JBI critical appraisal checklist, and the ROBINS-I tool are available in *Table S1*, *Table S2*, and *Table S3* respectively.

**Table 1:** Study details

Study title and procedure name	Reference	Year of first surgery	Year of first publication	Interval (years)	Follow-up (years)	Number of patients in first publication	Number of patients (total)/number of centres performing the operation	IRB or ethics committee approval	Trial registration at patient enrolment
Novel metabolic surgery: first Asia series and short-term results of laparoscopic proximal jejunal bypass with sleeve gastrectomy Combination of gastric restrictive surgery and JIB/modified JIB	^11^	2014	2016	2	0.5	65	65/1	No	No
Evaluation of the outcome of a proposed more physiological bypass surgery technique in morbid obesity: long term 3 years follows up Combination of gastric restrictive surgery and JIB/modified JIB	^12^	1999	2022	23	3	256	256/1	Yes	No
Modified jejunoileal bypass surgery with biliary diversion for morbid obesity and changes in liver histology during follow-up Combination of gastric restrictive surgery and JIB/modified JIB	^13^	1982	2007	25	5	43	45/1	No	No
Short-term outcomes of sleeve gastrectomy plus jejunojejunal bypass: a retrospective comparative study with sleeve gastrectomy and Roux-en-Y gastric bypass in Chinese patients with BMI ≥35 kg/m^2^ Combination of gastric restrictive surgery and JIB/modified JIB	^14^	2010	2019	9	1	83	83/1	Yes	No
Short-term outcomes of sleeve gastrectomy plus uncut jejunojejunal bypass (SG–uncut JJB) in patients with obesity: a preliminary prospective cohort study Combination of gastric restrictive surgery and JIB/modified JIB	^15^	2020	2023	3	1	21	21/1	Yes	Yes
Vertical isolated gastroplasty with gastro-enteral bypass: preliminary results Combination of gastric restrictive surgery and JIB/modified JIB	^16^	2004	2006	2	1	30	30/1	Yes	No
Histologic and microbiological findings of the defunctionalized loop in sleeve gastrectomy with jejunal bypass Combination of gastric restrictive surgery and JIB/modified JIB	^17^	2004	2021	17	NA	1074	1074/1	Yes	No
Comparative study of laparoscopic sleeve gastrectomy with or without jejunal bypass Combination of gastric restrictive surgery and JIB/modified JIB	^18^	2014	2024	10	3	82	122/1	Yes	No
Sleeve gastrectomy plus uncut jejunojejunal bypass for the treatment of obesity and type 2 diabetes Combination of gastric restrictive surgery and JIB/modified JIB	^19^	2019	2022	3	1	24	24/1	Yes	Yes
Isolated intestinal transit bipartition: a new strategy for staged surgery in superobesity Isolated transit bipartition	^20^	NA	2019	NA	1	41	41/1	Yes	No
Reoperation after loop duodenojejunal bypass with sleeve gastrectomy: a 9-year experience Modifications of SADI bypass	^21^	2011	2024	13	3	337	337/1	Yes	No
Laparoscopic duodenojejunal bypass with sleeve gastrectomy: preliminary results of a prospective series from India Modifications of SADI bypass	^22^	2008	2012	4	1.5	38	38/1	Yes	No
Short-term outcomes of laparoscopic sleeve gastrectomy with duodenojejunal bypass for morbid obesity Modifications of SADI bypass	^23^	2019	2020	1	0.5	56	56/1	Yes	No
Ileal food diversion: a simple, powerful and easily revisable and reversible single-anastomosis gastric bypass Modifications of SADI bypass	^24^	2009	2015	6	4	68	200/1	No	No
Factors predicting weight loss after “sleeve gastrectomy with loop duodenojejunal bypass” surgery for obesity Modifications of SADI bypass	^25^	2013	2020	7	3	126	131/1	Yes	No
Laparoscopic single-anastomosis duodenal-jejunal bypass with sleeve gastrectomy (SADJB-SG): surgical risk and long-term results Modifications of SADI bypass	^26^	2011	2019	8	5	148	148/1	Yes	No
Five-year-results of laparoscopic sleeve gastrectomy with duodenojejunal bypass for weight loss and type 2 diabetes mellitus Modifications of SADI bypass	^27^	2007	2017	10	5	120	120/1	Yes	No
Single-anastomosis sleeve jejunal bypass, a novel bariatric surgery, versus other familiar methods: results of a 6-month follow-up-a comparative study Modifications of SADI bypass	^28^	2016	2019	3	0.5	25	47/1	Yes	No
Effects of different metabolic states and surgical models on glucose metabolism and secretion of ileal L-cell peptides: results from the HIPER-1 study Sleeve gastrectomy with diverted transit bipartition	^29^	NA	2019	NA	2	60	60/1	Yes	Yes
Technical feasibility and safety profile of laparoscopic diverted sleeve gastrectomy with ileal transposition (DSIT) Sleeve gastrectomy with diverted transit bipartition	^30^	2011	2015	4	2	360	454/1	Yes	No
Laparoscopic ileal interposition with diverted sleeve gastrectomy for treatment of type 2 diabetes Sleeve gastrectomy with diverted transit bipartition	^31^	2010	2012	2	0.75	17	17/1	Yes	No
A novel weight-reducing operation: lateral subtotal gastrectomy with silastic ring plus small bowel reduction with omentectomy Sleeve gastrectomy with enterectomy	^32^	2000	2008	8	3	246	246/1	No	No
Comparative study of laparoscopic sleeve gastrectomy with and without partial enterectomy and omentectomy Sleeve gastrectomy with enterectomy	^33^	NA	2012	NA	1	40	40/1	Yes	No
Metabolic effects of an entero-omentectomy in mildly obese type 2 diabetes mellitus patients after three years Sleeve gastrectomy with enterectomy	^34^	2006	2011	5	3	9	9/1	Yes	No
Bariatric surgery in adolescents: preliminary 1-year results with a novel technique (Santoro III) Sleeve gastrectomy with enterectomy	^35^	2003	2010	7	1	10	12/1	Yes	No
Digestive adaptation with intestinal reserve: a neuroendocrine-based operation for morbid obesity Sleeve gastrectomy with enterectomy	^36^	2002	2003	1	1	3	486/1	Yes	No
The short-term effects of transit bipartition with sleeve gastrectomy and distal-Roux-en-Y gastric bypass on glycemic control, weight loss, and nutritional status in morbidly obese and type 2 diabetes mellitus patients Sleeve gastrectomy with transit bipartition *versus* distal RYGB	^37^	2013	2021	8	1	26	26/1	Yes (but in 2020)	No
Is transit bipartition a better alternative to biliopancreatic diversion with duodenal switch for superobesity? Comparison of the early results of both procedures Sleeve gastrectomy with transit bipartition VS DS	^38^	2017	2020	3	1	71	71/1	No	No
Laparoscopic ileal interposition with diverted sleeve gastrectomy versus laparoscopic transit bipartition with sleeve gastrectomy for better glycemic outcomes in T2DM patients Sleeve gastrectomy with transit bipartition *versus* ileal interposition	^39^	NA	2018	NA	1	48	48/1	Yes	No
Laparoscopic sleeve gastrectomy with ileal interposition (‘neuroendocrine brake’)—pilot study of a new operation Sleeve with ileal interposition	^40^	2003	2006	3	0.1	19	454/1	Yes	No
Laparoscopic Roux-en-Y gastric bypass with ileal transposition—an alternative surgical treatment for type 2 diabetes mellitus and gastroesophageal reflux Sleeve with ileal interposition	^41^	NA	2015	NA	0.75	1	1/1	No	No
Type 2 diabetes mellitus remission following laparoscopic sleeve gastrectomy and hindgut-based procedure: a retrospective multicenter study Sleeve with ileal interposition	^42^	2007	2024	17	5	62	62/3	Yes	No
Early results of laparoscopic sleeve gastrectomy with loop bipartition Transit bipartition with sleeve	^43^	2017	2018	1	1	22	22/1	No	No
Metabolic effects of sleeve gastrectomy with transit bipartition in obese females with type 2 diabetes mellitus: results after 1-year follow-up Transit bipartition with sleeve	^44^	2016	2018	2	1	35	35/1	Yes	No
A prospective randomized controlled trial of the metabolic effects of sleeve gastrectomy with transit bipartition Transit bipartition with sleeve	^45^	2014	2018	4	2	10	10/3	Yes	Yes
Laparoscopic sleeve gastrectomy with transit loop bipartition and transit bipartition in type 2 diabetic patients with obesity: a retrospective analysis Transit bipartition with sleeve	^46^	2017	2023	6	1	32	59/2	No	No
Comparative evaluation of efficiency for gastroileostomy anastomosis in laparoscopic transit bipartition with sleeve gastrectomy between linear and circular staplers Transit bipartition with sleeve	^47^	2018	2022	4	1	21	21/3	Yes	No
Pilot study of a new model of bariatric surgery: laparoscopic intestinal bipartition-safety and efficacy against metabolic disorders Transit bipartition with sleeve	^48^	2011	2018	7	5	7	7/1	Yes	No
Single anastomosis sleeve ileal bypass: new step in the evolution of bariatric surgeries Transit bipartition with sleeve	^49^	2014	2017	3	1	45	45/1	Yes	No
Sleeve gastrectomy with transit bipartition: a potent intervention for metabolic syndrome and obesity Transit bipartition with sleeve	^50^	2012	2012	0.1	5	1020	1020/1	Yes	No
The outcomes of single anastomosis sleeve jejunal bypass as a treatment for morbid obesity (two-year follow-up) Transit bipartition with sleeve	^51^	2016	2021	5	2	150	1986/1	Yes	No
Comparison of laparoscopic sleeve gastrectomy and single anastomosis sleeve ileal bypass in type 2 diabetes mellitus remission using international criteria Transit bipartition with sleeve	^52^	2015	2023	8	1	30	30/1	Yes	No
Evaluation of the efficacy of single anastomosis sleeve ileal (SASI) bypass for patients with morbid obesity: a multicenter study Transit bipartition with sleeve	^53^	2014	2020	5	1	605	679/10	Yes	No
Sleeve gastrectomy with transit bipartition in a series of 883 patients with mild obesity: early effectiveness and safety outcomes Transit bipartition with sleeve	^54^	2015	2022	7	1	883	883/2	Yes	No
Laparoscopic sleeve gastrectomy with loop bipartition: a novel metabolic operation in treating obese type II diabetes mellitusTransit bipartition with sleeve	^55^	2012	2014	2	1	1	1/1	No	No
Sleeve gastrectomy plus single anastomosis sleeve ileal bipartition versus sleeve gastrectomy alone: the role of bipartition Transit bipartition with sleeve	^56^	2021	2024	3	1	39	39/1	Yes	No
Sleeve gastrectomy with Braun anastomosis transit bipartition (B-TB): a potential midway between single anastomosis and Roux-en-Y transit bipartition Transit bipartition with sleeve	^57^	2020	2021	1	0.25	10	10/1	Yes	No
Sleeve gastrectomy plus side-to-side jejunoileal anastomosis for the treatment of morbid obesity and metabolic diseases: a promising operation Transit bipartition with sleeve	^58^	2008	2012	4	2	32	32/1	Yes	No
A simple food-diverting operation for type 2 diabetes treatment. preliminary results in humans with BMI 28–32 kg/m**2** Transit bipartition with sleeve	^59^	NA	2017	NA	2	6	6/3	Yes	Yes
Comparative study between laparoscopic sleeve gastrectomy and single anastomosis sleeve ileal bypass in management of morbid obese patients Modifications of SADI bypass	^60^	2022	2024	2	1	20	20/1	Yes	No
Efficacy of single anastomosis sleeve-ileal bypass in weight control and resolution of type 2 diabetes mellitus—a retrospective cohort study Modifications of SADI bypass	^61^	NA	2024	NA	1	31	31/2	Yes	No
Single anastomosis sleeve ileal bypass (SASI): a single-center initial report Modifications of SADI bypass	^62^	2020	2022	2	1	19	19/1	No	No
Single anastomosis sleeve ileal (SASI) bypass as a primary and revisional procedure: a single-centre experience Modifications of SADI bypass	^63^	2018	2023	5	2	15	15/1	Yes	No
Safety and efficacy of single anastomosis sleeve ileal (SASI) bypass surgery on obese patients with type ii diabetes mellitus during a one-year follow-up period: a single center cohort study Modifications of SADI bypass	^64^	2017	2023	6	1	61	61/1	No	No
One anastomosis transit bipartition (OATB) Transit bipartition with sleeve	^65^	2015	2023	8	5	68	68/1	Yes	No
A safety study of laparoscopic single-anastomosis duodeno-ileal bypass with gastric plication (SADI-GP) in the management of morbid obesity Modifications of SADI bypass	^66^	2018	2022	4	1	17	17/1	Yes	Yes
Medium-term results of combined laparoscopic sleeve gastrectomy and modified jejuno-ileal bypass in bariatric surgery Combination of gastric restrictive surgery and JIB/modified JIB	^67^	2004	2016	12	4	168	189/1	yes	no

IRB, Institutional Review Board; JIB, jejunoileal bypass; NA, not available; SADI, single-anastomosis duodenoileal; RYGB, Roux-en-Y gastric bypass; VS, versus; DS, duodenal switch.

#### NOS

The mean(s.d.) score was 5.0(1.4) out of 9. Out of the 57 studies, only 1 study was rated as high quality (score ≥7), while the majority (45 studies, 78%) were of very low to low quality. No study reported loss-to-follow-up rates, 15 of 57 (26%) studies had a follow-up interval ≥3 years, and 8 of 57 (14%) studies were conducted across multiple centres.

#### JBI critical appraisal checklist

There were frequent deficiencies in reporting follow-up duration (incomplete in 38 of 57 studies, 66%) and using standard objective criteria for measuring outcomes (inconsistent in 42 of 57 studies, 74%).

#### ROBINS-I tool

Out of the 57 studies, 56 (98%) were judged to have a serious overall risk of bias, with the single greatest contributor being bias due to confounding (D1). As most studies were single-arm case series, there was no comparison group, and thus no ability to control for confounding factors that influence outcomes.

### Timeline of innovation and publication

There was a significant delay between the first reported human operation and the first peer-reviewed publication, with a median publication lag of 5 (i.q.r. 3–8, range 0.1–25) years. When analysed by publication interval, only five studies (9%) were published before the introduction of the IDEAL framework in 2009, none of which reported prospective trial registration. Among the 52 studies published after 2009, only 6 (12%) reported prospective trial registration. Out of the 57 studies, 14 (25%) reported a lag of ≥8 years, indicating that, for a substantial proportion of innovations, outcomes were not disseminated to the wider community for nearly a decade.

### Patient numbers and follow-up

The median number of patients reported in the initial publication for a novel procedure was 39 (range 1–1074) patients. The cumulative number of patients reported across all publications for these procedures was 10 825, but long-term follow-up data were scarce. The median follow-up duration was 2 (i.q.r. 1–3) years. Only 15 studies (26%) reported outcomes at ≥3 years and only 8 studies (14%) reported outcomes at ≥5 years.

### Ethical oversight and transparency

Formal ethical oversight was inconsistent with reported IRB or ethics committee approval in 47 of the 57 studies (82%). Prospective registration in a clinical trials registry at the time of patient enrolment was confirmed in only six studies (11%).

## Discussion

This systematic review using non-standard intestinal-based MBS as a case example revealed a consistent and concerning pattern in surgical innovation: the widespread adoption of innovative techniques is occurring within a framework characterized by delayed publication, inadequate long-term data, and a profound lack of prospective trial registration. The finding that over 10 000 patients have undergone these procedures outside a robust, transparent research framework is a cause of serious concern. This MBS case study serves as a stark indicator of systemic failures that resonate across the broader landscape of surgical innovation. Comparable patterns have characterized other areas of surgical innovation: early liver transplantation and laparoscopic cholecystectomy spread rapidly before standardized oversight, and, more recently, robotic and endovascular techniques have demonstrated similar reporting delays and regulatory gaps.

The results stand in sharp contrast to the principles of the IDEAL framework^9^, which provides a recommended pathway for responsible surgical innovation. The review spans from 2000 to 2024, encompassing intervals both before and after the 2009 publication of the IDEAL framework. While expectations for prospective registration were less formalized earlier in this interval, the persistent lack of improvement after 2009 underscores a systemic failure to adopt evolving standards for responsible innovation. The IDEAL model proposes a staged progression from idea (stage 1) through development (stage 2a), exploration (stage 2b), assessment (stage 3), and long-term study (stage 4). The multi-year lag between first-in-human procedures and first publication, coupled with the initiation of studies on large patient cohorts without prospective registration, represents a collapse of this staged approach. Procedures are effectively leaping from early development (stage 2a) directly into a form of unregulated, large-scale practice, entirely bypassing the critical exploratory and assessment phases (stages 2b and 3) where safety and efficacy should be formally established against comparator treatments.

Several factors likely contribute to this phenomenon. A ‘pioneer’ mentality, financial incentives in certain practice settings, and academic pressure to innovate can inadvertently prioritize rapid adoption over methodical evaluation. While single-centre reports are appropriate and expected for early IDEAL stages (2a/2b), the systemic failure to subsequently organize collaborative, prospective studies (stage 3) signifies a breakdown in the innovation pipeline. This pattern of initial enthusiasm followed by a paucity of confirmatory evidence is not new. Mark Ravitch, father of modern paediatric surgery, observed: ‘In general, if a technique has been adequately reported in a major journal, but was then not persistently followed up by its creators with a succession of papers confirming the original success, and was not picked up by others, the conclusion may safely be drawn that the procedure was defective or unattractive, and had probably been tried by others, who found it wanting, perhaps without bothering even to publish’^68^. The findings, showing that many innovative MBS procedures were reported with initial cohorts but lacked long-term follow-up and widespread independent validation, resonate strongly with this decades-old wisdom. Furthermore, the role of journals must be scrutinized; the publication of studies on unregistered interventions involving hundreds of patients without long-term data, as seen in this cohort, sets a low standard for the entire community and fails to enforce existing ethical guidelines.

The ethical implications of these findings are paramount. Patients considering MBS may experience high levels of motivation and sometimes desperation, which can complicate fully informed decision-making when novel, poorly evidenced procedures are presented as treatment options^69^. Offering such interventions outside the context of a formally registered clinical trial challenges the very principles of informed consent and patient autonomy. The finding that 90% of these procedures were introduced without prospective registration suggests that thousands of patients may not have been fully aware of the experimental nature of treatments received, a situation exacerbated when patients are paying out of the pocket.

The issue of evidence quality in the field of obesity treatment has never been more critical. MBS now exists in a competitive therapeutic landscape alongside highly effective glucagon-like peptide-1 receptor agonists. The evidence base for these pharmacological therapies is generated through large, rigorous, industry-sponsored RCTs that are prospectively registered and meticulously reported. If the metabolic bariatric surgical community wishes to maintain credibility and defend the role played in treating obesity, this community must generate evidence of comparable robustness. The current practice of introducing techniques with low-quality data, as documented in this review, fundamentally undermines the field’s standing with patients, referring physicians, and policymakers. A structural and cultural change is urgently needed. Scientific societies like the IFSO must move beyond position statements and implement actionable policies. The IDEAL framework provides the best-available roadmap for responsible surgical innovation, but remains ineffective without mandatory adoption. Professional societies, journals, and regulators must enforce the principles of the IDEAL framework as non-negotiable requirements for innovation oversight including for example core outcome sets for the reporting of innovative MBS procedures, establishing oversight committees, mandatory registries for first-in-human cases and outcomes, and linking centre accreditation or surgeon certification to adherence to IDEAL principles. Similarly, journals have a responsibility to raise the bar. Journals should universally mandate prospective registration as a non-negotiable condition for publication for any study involving an interventional innovation according to current standards. By refusing to publish unregistered research, journals can become powerful enforcers of ethical standards.

This review has several limitations. First, the review focused on a defined cohort of intestinal-based procedures within MBS over a 25-year interval as a case example within surgical innovation, but the results may not be fully generalizable to all surgical innovations though the overarching themes are likely shared. Second, the exclusion of non-English literature may have introduced selection bias. Third, by relying on published reports, the study likely misses procedures that were abandoned after early, poor outcomes that went unpublished. However, this publication bias may underestimate the true scale of the problem. Fourth, the exclusion of device-based therapies was necessary to maintain a homogeneous cohort, but means these findings may not directly apply to innovations that follow a different regulatory pathway.

In conclusion, surgical innovation is essential for progress, but must be synonymous with scientific rigour, transparency, and commitment to patient safety. The introduction of non-standard MBS procedures, as illustrated by this cohort of intestinal-based operations, was characterized by delayed reporting, a near-total lack of prospective registration, and insufficient long-term evidence. This pattern persisted even after the 2009 introduction of the IDEAL framework. This model is ethically questionable and scientifically unsound, exposing thousands of patients to potential harm, and damages the credibility of the entire surgical field. The experience of MBS provides lessons relevant across all surgical disciplines, emphasizing transparency, evidence generation, and patient safety as shared imperatives.

## Supplementary Material

znag047_Supplementary_Data

## Data Availability

The data that support the findings of this study are available on request from the corresponding author.
