# Supplementary material for: Navigating uncharted territory in surgical innovation: systematic review of non-standard metabolic bariatric surgery procedures
Source: Br J Surg. 2026 Apr 16;113(5):znag047. doi: 10.1093/bjs/znag047 (PMC13177703; doi:10.1093/bjs/znag047)
Supplement: znag047_Supplementary_Data [file znag047_supplementary_data.docx]

**Navigating Uncharted Territory in Surgical Innovation: A systematic review of non-standard Metabolic Bariatric Surgery procedures**

**Supplementary Materials - Index**

**Supplementary Methods**

Search strategy *page 4*

Eligibility criteria   *page 4*

Data extraction & synthesis   *page 4*

Quality assessment *page 4*

Protocol registration *page 4*

**Supplementary Results**

Procedure classification   *page 5*

**Supplementary Appendixes**

PRISMA 2020 checklist *page 8*

PRISMA 2020 abstract checklist  *page 10*

**Supplementary Figures and| Tables**

Table S1: NOS assessment *page 13*

Table S2: JBI assessment *page 20*

Table S3: ROBINS-I assessment *page 26*

**References**  *page 34*|

**Supplementary Methods**

**1. Search strategy**
A systematic search was performed in PubMed, Embase, Scopus, Cochrane Library, and Web of Science for studies published between January 2000 and December 2024. The Boolean search combined controlled vocabulary and free-text terms for bariatric OR metabolic surgery AND (unlisted OR innovative OR novel OR new technique OR modification). No language restrictions were applied. References of relevant articles were hand-searched to identify additional records.

**2. Eligibility criteria**
Studies were included if they:

- Reported first-in-human or early clinical series of non-standard primary metabolic or bariatric operations involving major intestinal modification.
- Included adult human participants (≥18 years).
- Reported at least one primary outcome of safety, efficacy, or procedural feasibility.

Exclusion criteria were:

- Revisional, purely restrictive, or device-based procedures.
- Animal or cadaver studies.
- Conference abstracts, editorials, or reviews.

**3. Data extraction and synthesis**
From each study, the following data were extracted: year of publication, country, sample size, follow-up duration, number of centres, type of procedure, oversight variables (IRB approval, registry registration), and quality-assessment scores. Data extraction was performed independently by two reviewers, and discrepancies were resolved by consensus. Given clinical and methodological heterogeneity, results were synthesised descriptively, reporting medians and interquartile ranges for continuous data.

**4. Quality assessment**
Methodological quality was evaluated using the Newcastle–Ottawa Scale (NOS) for cohort studies, the Joanna Briggs Institute checklist for case series, and ROBINS-I for non-randomised studies. Studies were classified as low, moderate, or high/serious risk of bias based on consensus scoring.

**5. Protocol registration and reporting**
This review followed the PRISMA 2020 statement and was prospectively registered in PROSPERO (CRD420250641346). The PRISMA flow diagram summarising study selection is provided in Figure S1. All extracted data and individual quality-assessment tables are available in Tables S1–S3.

**Supplementary Results**

**Classification of Non-Standard Intestinal-Based Metabolic and Bariatric Surgery Procedures**

The 57 studies included in this systematic review described a diverse array of innovative procedures that were not endorsed by major international guidelines (IFSO/ASMBS) at the time of their introduction. For descriptive purposes, these procedures were categorized into five main groups based on their anatomical and functional principles.

**1. Combination of Gastric Restrictive Surgery and Jejunoileal Bypass (JIB) / Modified JIB**
This category includes procedures that combine a sleeve gastrectomy (or another form of gastric restriction) with variants of the JIB.

- *Examples from the dataset:*
  - Laparoscopic Proximal Jejunal Bypass with Sleeve Gastrectomy
  - Sleeve Gastrectomy plus Jejunojejunal Bypass (SG-JJB)
  - Sleeve Gastrectomy plus Uncut Jejunojejunal Bypass (SG-uncut JJB)
  - Vertical Isolated Gastroplasty with Gastro-enteral Bypass
  - Modified Jejunoileal Bypass with Biliary Diversion
  - Laparoscopic Sleeve Gastrectomy with Jejunal Bypass
  - Combined Laparoscopic Sleeve Gastrectomy and Modified Jejuno-Ileal Bypass

**2. Modifications of the Single-Anastomosis Duodeno-Ileal Bypass (SADI)**
This group comprises procedures that are technical variations of the SADI-S or loop duodenojejunal bypass, characterized by a single anastomosis connecting the duodenum or proximal jejunum to the ileum, combined with a sleeve gastrectomy.

- *Examples from the dataset:*
  - Loop Duodenojejunal Bypass with Sleeve Gastrectomy (LDJB-SG)
  - Single-Anastomosis Duodeno-Jejunal Bypass with Sleeve Gastrectomy (SADJB-SG)
  - Single-Anastomosis Sleeve Jejunal Bypass
  - Ileal Food Diversion (a single-anastomosis gastric bypass variant)
  - Single-Anastomosis Duodeno-Ileal Bypass with Gastric Plication (SADI-GP)

**3. Sleeve Gastrectomy with Transit Bipartition and its Variants**
This is a broad category centered on the principle of "transit bipartition," where ingested food travels through two pathways: one through the natural duodenal route and another through a gastro-ileal or gastro-jejunal anastomosis.

*Examples from the dataset:*

- Sleeve Gastrectomy with Transit Bipartition (SG-TB)
- Single Anastomosis Sleeve Ileal (SASI) Bypass
- Laparoscopic Intestinal Bipartition
- Sleeve Gastrectomy with Loop Bipartition
- One Anastomosis Transit Bipartition (OATB)
- Sleeve Gastrectomy with Braun Anastomosis Transit Bipartition (B-TB)
- Sleeve Gastrectomy plus Side-to-Side Jejunoileal Anastomosis

**4. Sleeve Gastrectomy with Ileal Interposition**
This category involves the transposition of a segment of the distal ileum to a more proximal location (typically near the duodenojejunal flexure).

- *Examples from the dataset:*
  - Sleeve Gastrectomy with Ileal Interposition
  - Diverted Sleeve Gastrectomy with Ileal Transposition (DSIT)
  - Laparoscopic Roux-en-Y Gastric Bypass with Ileal Transposition

**5. Sleeve Gastrectomy with Enterectomy**
This group includes procedures that combine that combine a sleeve gastrectomy with the resection of a portion of the small bowel and/or omentum.

- *Examples from the dataset:*
  - Sleeve Gastrectomy with Partial Enterectomy and Omentectomy
  - Lateral Subtotal Gastrectomy with Silastic Ring plus Small Bowel Reduction with Omentectomy
  - Digestive Adaptation with Intestinal Reserve (Santoro III procedure)
  - Entero-omentectomy

**Note on Comparative Studies:**
Several included studies were comparative in nature, evaluating one of the above-listed innovative procedures against another or against a standard procedure (e.g., Sleeve Gastrectomy with Transit Bipartition vs. Sleeve with Ileal Interposition; SASI vs. Sleeve Gastrectomy). For the purpose of this review, the data pertaining to the innovative arm(s) of these studies were included in the analysis.

**Supplementary Appendixes**

**Supplementary material 3. PRISMA 2020 Checklist for Systematic Reviews- Manuscript (1)**

| **Section** | **Item #** | **PRISMA 2020 Item** | **Reported (Yes/No)** | **Page/Line in Manuscript** |
| --- | --- | --- | --- | --- |
| **TITLE** | 1 | Identify the report as a systematic review. | Yes | Title: "A systematic review of non-standard Metabolic Bariatric Surgery procedures" |
| **ABSTRACT** | 2 | Provide a structured abstract. | Yes | Abstract (Background, Methods, Results, Conclusion) |
| **INTRODUCTION** | 3 | Describe the rationale for the review. | Yes | Introduction (par. 1–4) |
|  | 4 | State the objectives and research question. | Yes | Introduction (final par.) |
| **METHODS** | 5 | Indicate whether a review protocol exists and where it can be accessed. | Yes | Methods: "prospectively registered in PROSPERO (CRD420250641346)" |
|  | 6 | Specify eligibility criteria. | Yes | Methods: Eligibility Criteria |
|  | 7 | Describe information sources and search strategy. | Yes | Methods: Search strategy and reference to PROSPERO |
|  | 8 | Describe study selection process. | Yes | Methods: Study selection and data extraction |
|  | 9 | Describe data extraction process. | Yes | Methods: Data extraction |
|  | 10 | List and define outcomes. | Yes | Methods: Key extracted variables listed |
|  | 11 | Describe risk of bias assessment. | Yes | Methods: Quality assessment |
|  | 12 | Describe synthesis methods. | Yes | Methods: Data synthesis |
| **RESULTS** | 13 | Describe study selection results (flow diagram). | Yes | Figure 1 (PRISMA flowchart) |
|  | 14 | Describe study characteristics. | Yes | Results: Study characteristics |
|  | 15 | Present risk of bias assessments. | Yes | Results: Methodological quality |
|  | 16 | Present results for each outcome. | Yes | Results: Timeline, Patient numbers, Ethical oversight |
| **DISCUSSION** | 17 | Summarize main findings. | Yes | Discussion (par. 1) |
|  | 18 | Discuss limitations. | Yes | Limitations section |
|  | 19 | Provide general interpretation. | Yes | Discussion and conclusion |
| **OTHER** | 20 | Describe funding sources. | Yes | Conflicts of Interest declared; no external funding |
|  | 21 | Declare conflicts of interest. | Yes | Conflicts of Interest explained |

**PRISMA 2020 Checklist for Abstracts of Systematic Reviews**

| Section/Topic | Item # | PRISMA-A Checklist Item | Reported? (Yes/No) | Location in Abstract |
| --- | --- | --- | --- | --- |
| **TITLE** | 1 | Identify the report as a systematic review. | **Yes** | "A systematic review of non-standard Metabolic Bariatric Surgery procedures" |
| **BACKGROUND** | 2 | Provide an explicit statement of the main objective(s) or question(s) the review addresses. | **Yes** | "...to evaluate reporting standards and ethical oversight for novel procedures." |
| **METHODS** | 3 | Specify the inclusion and exclusion criteria for the review. | **Yes** | "Inclusion criteria: original studies reporting on non-standard primary MBS procedures involving significant intestinal modification... Exclusion criteria: reviews, editorials, conference abstracts, revisional surgery, purely restrictive procedures, and device-based interventions." |
| **METHODS** | 4 | Specify the information sources (e.g., databases, registers) used to identify studies. | **Yes** | "PubMed/MEDLINE, Web of Science, Scopus, Cochrane Library, and Embase" |
| **METHODS** | 5 | Specify the date when the search was conducted (or the date range). | **Yes** | "from January 2000 to December 2024" |
| **METHODS** | 6 | Specify the methods used to assess risk of bias in the included studies. | **Yes** | "assessed risk of bias using the Newcastle-Ottawa Scale, Joanna Briggs Institute checklist, and ROBINS-I tool." |
| **RESULTS** | 7 | Give the total number of included studies and participants. | **Yes** | "57 included studies (10,754 patients)" |
| **RESULTS** | 8 | Present summary measures for primary outcomes (e.g., median, IQR). | **Yes** | "median time from first human operation to publication was 5 years (IQR 3-8)... median initial cohort size was 39 patients (range 1-1074)..." |
| **RESULTS** | 9 | Describe the direction of the effect (e.g., benefit, harm) if meta-analysis was conducted. | **N/A** | Meta-analysis was not performed. |
| **DISCUSSION** | 10 | Provide a brief summary of the strengths and limitations of the evidence. | **Partial** | Limitations are implied in findings (e.g., high risk of bias, lack of registration). Strengths not explicitly stated in abstract. |
| **DISCUSSION** | 11 | Provide a general interpretation of the results in the context of other evidence. | **Yes** | "These findings highlight a systemic failure in surgical innovation governance..." |
| **OTHER** | 12 | Specify the funding source(s) for the systematic review. | **Yes** | Conflicts of Interest statement implies no external funding. |
| **OTHER** | 13 | Provide the register and registration number for the review. | **Yes** | PROSPERO registration number (CRD420250641346) is included in the abstract. |

**Supplementary Figures and Tables**

**Table S1. Newcastle–Ottawa Scale Assessment (2)**

**Interpretation Guide**

The NOS is formally for studies with a comparison group. Most studies here are single-arm case series. For these, the "Selection of the Non-Exposed Cohort" (S2) and the entire "Comparability" domain (C1, C2) are not applicable, making their maximum possible score 7/9.

NOS Score Range Number of Studies Percentage of Total

Very Low (0-2) 3 5.2%

Low (3-4) 42 72.4%

Moderate (5-6) 12 20.7%

High (7-9), 1 1.7%

Major Weakness: Not a single study in the dataset provided information on loss-to-follow-up rates (O3).

Limited Long-Term Data: Only about a third of studies had a follow-up period long enough (≥3 years) to be confident in the durability of outcomes.

Limited Generalizability: Only 10% of studies were conducted across multiple centers, meaning most findings are from single institutions and may not be widely applicable.

| Study Name / Identifier | S1 | S2 | S3 | S4 | C1 | C2 | O1 | O2 | O3 | **Total** | Notes |
| --- | --- | --- | --- | --- | --- | --- | --- | --- | --- | --- | --- |
| **Cohort A: Likely Comparative Studies** |  |  |  |  |  |  |  |  |  |  |  |
| Liang (2019) | ★ | ☆ | ☆ | ★ | ☆ | ★ | ★ | ★ | ★ | **4/9** | Retrospective comparative study. |
| Zhao (2024) | ★ | ☆ | ☆ | ★ | ☆ | ★ | ★ | ☆ | ★ | **5/9** | Comparative study. 3y fu is adequate. |
| Yormaz (2018) | ★ | ☆ | ☆ | ★ | ☆ | ★ | ★ | ★ | ★ | **4/9** | Comparative study (ileal interp. vs. bipartition). |
| Cagiltay (2019) | ★ | ☆ | ☆ | ★ | ☆ | ★ | ★ | ☆ | ★ | **5/9** | RCT design is a major strength (not fully captured). |
| Wang (2012) - Comp. | ★ | ☆ | ☆ | ★ | ☆ | ★ | ★ | ★ | ★ | **4/9** | Comparative study. |
| Topart (2020) | ★ | ☆ | ☆ | ★ | ☆ | ★ | ★ | ★ | ★ | **4/9** | Comparative study (Transit Bipartition vs. DS). |
| Sisik (2023) | ★ | ☆ | ☆ | ★ | ☆ | ★ | ★ | ★ | ★ | **4/9** | Comparative study. |
| Emile (2019) | ☆ | ☆ | ☆ | ★ | ☆ | ★ | ★ | ★ | ★ | **5/9** | Multicenter (10), large N. Comparative? |
| Lee (2024) | ★ | ☆ | ☆ | ★ | ☆ | ★ | ★ | ★ | ★ | **4/9** | Comparative study. |
| Caramelli (2018) | ★ | ☆ | ☆ | ★ | ☆ | ☆ | ★ | ☆ | ★ | **5/9** | **RCT.** Major strength. |
| Shebl (2022) | ★ | ☆ | ☆ | ★ | ☆ | ★ | ★ | ★ | ★ | **4/9** | Comparative study. |
| Jaworski (2024) | ★ | ☆ | ☆ | ★ | ☆ | ★ | ★ | ★ | ★ | **4/9** | Comparative study. |
| **Cohort B: Single-Arm Case Series (No Comparison Group)** |  |  |  |  |  |  |  |  |  |  |  |
| Huang (2016) | ★ | ★ | ☆ | ★ | ★ | ★ | ★ | ★ | ★ | **2/7** | Short-term (0.5y). |
| Elbanna (2022) | ★ | ★ | ☆ | ★ | ★ | ★ | ★ | ☆ | ★ | **3/7** | Long interval to publication (23y). |
| Fazel (2007) | ★ | ★ | ☆ | ★ | ★ | ★ | ★ | ☆ | ★ | **3/7** | Historical series (25y interval). |
| Han (2023) | ★ | ★ | ☆ | ★ | ★ | ★ | ★ | ★ | ★ | **2/7** | Prospective is a strength, but short-term. |
| Alamo (2006) | ★ | ★ | ☆ | ★ | ★ | ★ | ★ | ★ | ★ | **2/7** | Single-center, small N. |
| Sepulveda (2021) | ★ | ★ | ☆ | ★ | ★ | ★ | ★ | N/A | ★ | **2/7** | Focus on histology, not primary outcomes. |
| Wang (2022) | ★ | ★ | ☆ | ★ | ★ | ★ | ★ | ★ | ★ | **2/7** | Single-center, small N. |
| Paiva de Godoy (2019) | ★ | ★ | ☆ | ★ | ★ | ★ | ★ | ★ | ★ | **2/7** | No surgery date. |
| Huang (2024) | ★ | ★ | ☆ | ★ | ★ | ★ | ★ | ☆ | ★ | **3/7** | Large N, long-term (9y) on reoperations. |
| Raj (2012) | ★ | ★ | ☆ | ★ | ★ | ★ | ★ | ★ | ★ | **2/7** | Prospective series is a strength. |
| Choi (2020) | ★ | ★ | ☆ | ★ | ★ | ★ | ★ | ★ | ★ | **2/7** | Very short-term (0.5y). |
| Greco (2015) | ★ | ★ | ☆ | ★ | ★ | ★ | ★ | ☆ | ★ | **3/7** | Long fu (4y) is a strength. |
| Vennapusa (2020) | ★ | ★ | ☆ | ★ | ★ | ★ | ★ | ☆ | ★ | **3/7** | Focus on predictive factors. |
| Lee (2019) | ★ | ★ | ☆ | ★ | ★ | ★ | ★ | ☆ | ★ | **3/7** | Long-term (5y) is a strength. |
| Seki (2017) | ★ | ★ | ☆ | ★ | ★ | ★ | ★ | ☆ | ★ | **3/7** | 5-year outcomes are a strength. |
| Rezaei (2019) | ★ | ★ | ☆ | ★ | ★ | ★ | ★ | ★ | ★ | **2/7** | Very short-term (0.5y), small N. |
| Celik (2015) | ★ | ★ | ☆ | ★ | ★ | ★ | ★ | ☆ | ★ | **3/7** | Large N, 2y fu. |
| Kota (2012) | ★ | ★ | ☆ | ★ | ★ | ★ | ★ | ★ | ★ | **2/7** | Very short-term fu (0.75y). |
| Heap (2008) | ★ | ★ | ☆ | ★ | ★ | ★ | ★ | ☆ | ★ | **3/7** | No IRB. Long interval to publication. |
| Lacombe (2011) - Clin Surg | ★ | ★ | ☆ | ★ | ★ | ★ | ★ | ☆ | ★ | **3/7** | Very small N (9). |
| Velhote (2010) | ★ | ★ | ☆ | ★ | ★ | ★ | ★ | ★ | ★ | **2/7** | Focus on adolescents. Small N. |
| Santoro (2003) | ★ | ★ | ☆ | ★ | ★ | ★ | ★ | - | ★ | **2/7** | Seminal paper. Small initial N. |
| Ece (2021) | ★ | ★ | ☆ | ★ | ★ | ★ | ★ | ★ | ★ | **2/7** | IRB approved late. |
| De Paula (2006) | ★ | ★ | ☆ | ★ | ★ | ★ | ★ | ★ | ★ | **2/7** | Pioneering paper. Very short-term fu (0.1y). |
| Ersoz (2015) | ★ | ★ | ☆ | ★ | ★ | ★ | ★ | ★ | ★ | **2/7** | N=1 (case report). No IRB. |
| Foschi (2024) | ☆ | ★ | ☆ | ★ | ★ | ★ | ★ | ☆ | ★ | **4/7** | Multicenter, long-term (5y). Low N at 5y fu. |
| Arslan (2018) | ★ | ★ | ☆ | ★ | ★ | ★ | ★ | ★ | ★ | **2/7** | No IRB, no registration. |
| Bilecik (2018) | ★ | ★ | ☆ | ★ | ★ | ★ | ★ | ★ | ★ | **2/7** | Specific population. |
| Demir (2023) | ★ | ★ | ☆ | ★ | ★ | ★ | ★ | ★ | ★ | **2/7** | No IRB. Multicenter. |
| Gulaydin (2022) | ★ | ★ | ☆ | ★ | ★ | ★ | ★ | ★ | ★ | **2/7** | Focuses on technical comparison. |
| Kassir (2018) | ★ | ★ | ☆ | ★ | ★ | ★ | ★ | ☆ | ★ | **3/7** | Very small N (7), long-term (5y) fu. |
| Salama (N/A) | ★ | ★ | ☆ | ★ | ★ | ★ | ★ | ★ | ★ | **2/7** | Critical data missing. |
| Santoro (2012) | ★ | ★ | ☆ | ★ | ★ | ★ | ★ | ☆ | ★ | **3/7** | Very large N (1020), long-term (5y). |
| Sewefi (2021) | ★ | ★ | ☆ | ★ | ★ | ★ | ★ | ☆ | ★ | **3/7** | Large N, 2y fu. |
| Mui (2014) | ★ | ★ | ☆ | ★ | ★ | ★ | ★ | ★ | ★ | **2/7** | N=1 (case report). No IRB. |
| Zhu (2021) | ★ | ★ | ☆ | ★ | ★ | ★ | ★ | - | ★ | **2/7** | Small N (10). No fu duration reported. |
| Melissas (2012) | ★ | ★ | ☆ | ★ | ★ | ★ | ★ | ☆ | ★ | **3/7** | 2y fu. |
| Melissas (2016) | ★ | ★ | ☆ | ★ | ★ | ★ | ★ | ☆ | ★ | **3/7** | Small N (6), registered. |
| Barski (2022) | ★ | ★ | ☆ | ★ | ★ | ★ | ★ | ★ | ★ | **2/7** | Informed consent "day before" is a concern. |
| Dowgiallo (2023) | ★ | ★ | ☆ | ★ | ★ | ★ | ★ | ☆ | ★ | **3/7** | Includes revisional procedures. |
| Alamdari (2023) | ★ | ★ | ☆ | ★ | ★ | ★ | ★ | ★ | ★ | **2/7** | No IRB. |
| Rossoni (2024) | ★ | ★ | ☆ | ★ | ★ | ★ | ★ | ☆ | ★ | **3/7** | Long-term (5y) results. |
| Balint (2022) | ★ | ★ | ☆ | ★ | ★ | ★ | ★ | ★ | ★ | **2/7** | Small N, registered. |
| Luhmann (2018) | ☆ | ★ | ☆ | ★ | ★ | ★ | ★ | ☆ | ★ | **4/7** | **Very long-term follow-up (8y).** Multicenter. |

**Table S2. JBI Critical Appraisal for Case Series - Detailed Study Assessment (3)**

| Study Name | Q1. Clear Inclusion Criteria? | Q2. Standard Condition Measurement? | Q3. Valid Identification? | Q4. Consecutive Inclusion? | Q5. Complete Inclusion? | Q6. Demographics Reported? | Q7. Clinical Info Reported? | Q8. Outcomes Clearly Reported? | Q9. Site Demographics Reported? | Q10. Appropriate Stats? | **Overall Summary & Key Critiques from Metadata** |
| --- | --- | --- | --- | --- | --- | --- | --- | --- | --- | --- | --- |
| **Huang (2016)** | Unclear | Yes | Yes | Unclear | Unclear | Partial | Partial | Yes | Yes | Unclear | Single-center, short-term (0.5y). No IRB, no trial reg. High risk of bias. |
| **Elbanna (2022)** | Unclear | Yes | Yes | Unclear | Unclear | Partial | Partial | Yes | Yes | Unclear | Very long interval (23y) between first surgery and publication. High risk of recall/loss-to-fu bias. |
| **Fazel (2007)** | Unclear | Yes | Yes | Unclear | Unclear | Partial | Partial | Yes | Yes | Unclear | Historical series (25y interval). No IRB. Very high risk of bias. |
| **Liang (2019)** | Unclear | Yes | Yes | Unclear | Unclear | Partial | Partial | Yes | Yes | Unclear | Comparative study design is a strength, but not captured fully here. |
| **Han (2023)** | Unclear | Yes | Yes | Unclear | Unclear | Partial | Partial | Yes | Yes | Unclear | Prospective cohort is a strength. Registered. Lower risk of bias. |
| **Alamo (2006)** | Unclear | Yes | Yes | Unclear | Unclear | Partial | Partial | Yes | Yes | Unclear | Single-center, small N. Short-term fu. |
| **Sepulveda (2021)** | Unclear | Yes | Yes | Unclear | Unclear | Partial | Partial | N/A | Yes | Unclear | Focus on histology/microbiology, not primary clinical outcomes. |
| **Zhao (2024)** | Unclear | Yes | Yes | Unclear | Unclear | Partial | Partial | Yes | Yes | Unclear | Comparative study, includes revisional cases. 3y fu is a strength. |
| **Wang (2022)** | Unclear | Yes | Yes | Unclear | Unclear | Partial | Partial | Yes | Yes | Unclear | Single-center, small N. Registered. |
| **Paiva de Godoy (2019)** | **No** | Yes | Yes | Unclear | Unclear | Partial | Partial | Yes | Yes | Unclear | **No date of first surgery provided.** Cannot assess timeline or evolution. |
| **Huang (2024)** | Unclear | Yes | Yes | Unclear | Unclear | Partial | Partial | Yes | Yes | Unclear | Large N, long-term (9y) experience focused on reoperations. Valuable for safety. |
| **Raj (2012)** | Unclear | Yes | Yes | Unclear | Unclear | Partial | Partial | Yes | Yes | Unclear | Prospective series is a strength. |
| **Choi (2020)** | Unclear | Yes | Yes | Unclear | Unclear | Partial | Partial | Yes | Yes | Unclear | Very short-term outcomes (0.5y). |
| **Greco (2015)** | Unclear | Yes | Yes | Unclear | Unclear | Partial | Partial | Yes | Yes | Unclear | No IRB. Long fu (4y) is a strength. |
| **Vennapusa (2020)** | Unclear | Yes | Yes | Unclear | Unclear | Partial | Partial | Yes | Yes | Unclear | Focus on predictive factors, which is a strength. |
| **Lee (2019)** | Unclear | Yes | Yes | Unclear | Unclear | Partial | Partial | Yes | Yes | Unclear | Long-term (5y) results are a significant strength. |
| **Seki (2017)** | Unclear | Yes | Yes | Unclear | Unclear | Partial | Partial | Yes | Yes | Unclear | 5-year outcomes for diabetes and weight. Strong contributor. |
| **Rezaei (2019)** | Unclear | Yes | Yes | Unclear | Unclear | Partial | Partial | Yes | Yes | Unclear | Very short-term (0.5y), small N. |
| **Cagiltay (2019)** | **No** | Yes | Yes | Unclear | Unclear | Partial | Partial | Yes | Yes | Unclear | **No date of first surgery.** RCT design is a major strength not reflected in this sheet. |
| **Celik (2015)** | Unclear | Yes | Yes | Unclear | Unclear | Partial | Partial | Yes | Yes | Unclear | Large N, 2y fu. |
| **Kota (2012)** | Unclear | Yes | Yes | Unclear | Unclear | Partial | Partial | Yes | Yes | Unclear | Very short-term fu (0.75y). |
| **Heap (2008)** | Unclear | Yes | Yes | Unclear | Unclear | Partial | Partial | Yes | Yes | Unclear | No IRB. Long interval to publication. |
| **Wang (2012)** | **No** | Yes | Yes | Unclear | Unclear | Partial | Partial | Yes | Yes | Unclear | **No date of first surgery.** |
| **Lacombe (2011)** | Unclear | Yes | Yes | Unclear | Unclear | Partial | Partial | Yes | Yes | Unclear | Very small N (9). |
| **Velhote (2010)** | Unclear | Yes | Yes | Unclear | Unclear | Partial | Partial | Yes | Yes | Unclear | Focus on adolescents. Small N. |
| **Lacombe (2011) - Clin Surg** | **No** | Yes | Yes | Unclear | Unclear | Partial | Partial | Yes | Yes | Unclear | **No date of first surgery.** Appears to be a duplicate/similar to above. |
| **Santoro (2003)** | Unclear | Yes | Yes | Unclear | Unclear | Partial | Partial | Yes | Yes | Unclear | Seminal paper for the technique. Small initial N with subsequent large reports. |
| **Ece (2021)** | Unclear | Yes | Yes | Unclear | Unclear | Partial | Partial | Yes | Yes | Unclear | IRB approved late (2020 for 2013 start). |
| **Topart (2020)** | Unclear | Yes | Yes | Unclear | Unclear | Partial | Partial | Yes | Yes | Unclear | Comparative study. No IRB. |
| **Yormaz (2018)** | **No** | Yes | Yes | Unclear | Unclear | Partial | Partial | Yes | Yes | Unclear | **No date of first surgery.** Comparative study. |
| **De Paula (2006)** | Unclear | Yes | Yes | Unclear | Unclear | Partial | Partial | Yes | Yes | Unclear | Pioneering paper. Very short-term fu (0.1y). |
| **Ersoz (2015)** | **No** | Yes | Yes | Unclear | Unclear | Partial | Partial | Yes | Yes | Unclear | **No date of first surgery.** No IRB. N=1 (case report). |
| **Foschi (2024)** | Unclear | Yes | Yes | Unclear | Unclear | Partial | Partial | Yes | Yes | Unclear | Multicenter, long-term (5y). Low N at 5y fu (20/62). |
| **Arslan (2018)** | Unclear | Yes | Yes | Unclear | Unclear | Partial | Partial | Yes | Yes | Unclear | No IRB, no registration. |
| **Bilecik (2018)** | Unclear | Yes | Yes | Unclear | Unclear | Partial | Partial | Yes | Yes | Unclear | Single-center, specific population (obese females with T2DM). |
| **Caramelli (2018)** | Unclear | Yes | Yes | Unclear | Unclear | Partial | Partial | Yes | Yes | Unclear | **RCT design** is a major strength. Registered. |
| **Demir (2023)** | Unclear | Yes | Yes | Unclear | Unclear | Partial | Partial | Yes | Yes | Unclear | No IRB. Multicenter. |
| **Gulaydin (2022)** | Unclear | Yes | Yes | Unclear | Unclear | Partial | Partial | Yes | Yes | Unclear | Focuses on technical comparison. |
| **Kassir (2018)** | Unclear | Yes | Yes | Unclear | Unclear | Partial | Partial | Yes | Yes | Unclear | Very small N (7), long-term (5y) fu. |
| **Salama** | **No** | Yes | Yes | Unclear | Unclear | Partial | Partial | Unclear | Yes | Unclear | **Critical data missing:** No surgery date, no publication year. |
| **Santoro (2012)** | Unclear | Yes | Yes | Unclear | Unclear | Partial | Partial | Yes | Yes | Unclear | Very large N (1020), long-term (5y). Major contributor. |
| **Sewefi (2021)** | Unclear | Yes | Yes | Unclear | Unclear | Partial | Partial | Yes | Yes | Unclear | Large N, 2y fu. |
| **Sisik (2023)** | Unclear | Yes | Yes | Unclear | Unclear | Partial | Partial | Yes | Yes | Unclear | Comparative study. |
| **Emile (2019)** | Unclear | Yes | Yes | Unclear | Unclear | Partial | Partial | Yes | Yes | Unclear | **Multicenter (10), large N (605).** Strong evidence for short-term safety/efficacy. |
| **Taskin (2022)** | Unclear | Yes | Yes | Unclear | Unclear | Partial | Partial | Yes | Yes | Unclear | **Very large N (883).** Multicenter. Major contributor. |
| **Mui (2014)** | Unclear | Yes | Yes | Unclear | Unclear | Partial | Partial | Yes | Yes | Unclear | N=1 (case report). No IRB. |
| **Lee (2024)** | Unclear | Yes | Yes | Unclear | Unclear | Partial | Partial | Yes | Yes | Unclear | Comparative study. |
| **Zhu (2021)** | Unclear | Yes | Yes | Unclear | Unclear | Partial | Partial | Unclear | Unclear | Unclear | Small N (10). No follow-up duration reported. |
| **Melissas (2012)** | Unclear | Yes | Yes | Unclear | Unclear | Partial | Partial | Yes | Yes | Unclear |  |
| **Melissas (2016)** | **No** | Yes | Yes | Unclear | Unclear | Partial | Partial | Yes | Yes | Unclear | **No date of first surgery.** Small N (6), registered. |
| **Shebl (2022)** | **No** | Yes | Yes | Unclear | Unclear | Partial | Partial | Yes | Yes | Unclear | **No date of first surgery.** |
| **Jaworski (2024)** | **No** | Yes | Yes | Unclear | Unclear | Partial | Partial | Yes | Yes | Unclear | **No date of first surgery.** Multicenter. |
| **Barski (2022)** | Unclear | Yes | Yes | Unclear | Unclear | Partial | Partial | Yes | Yes | Unclear | No IRB. Informed consent "day before surgery" is a potential ethical concern. |
| **Dowgiallo (2023)** | Unclear | Yes | Yes | Unclear | Unclear | Partial | Partial | Yes | Yes | Unclear | Includes revisional procedures. |
| **Alamdari (2023)** | Unclear | Yes | Yes | Unclear | Unclear | Partial | Partial | Yes | Yes | Unclear | No IRB. |
| **Rossoni (2024)** | Unclear | Yes | Yes | Unclear | Unclear | Partial | Partial | Yes | Yes | Unclear | Long-term (5y) results. |
| **Balint (2022)** | Unclear | Yes | Yes | Unclear | Unclear | Partial | Partial | Yes | Yes | Unclear | Small N, registered. |
| **Luhmann (2018)** | Unclear | Yes | Yes | Unclear | Unclear | Partial | Partial | Yes | Yes | Unclear | **Very long-term follow-up (8y).** Multicenter. |

**Table S3. Risk Of Bias In Non-randomized Studies - of Interventions (ROBINS-I) (4)**

| Study Name | D1 | D2 | D3 | D4 | D5 | D6 | D7 | **Overall Risk of Bias** | **Justification for Overall Judgement** |
| --- | --- | --- | --- | --- | --- | --- | --- | --- | --- |
| **Huang (2016)** | S | M | L | L | S | M | NI | **Serious** | Single-arm case series with no control for confounding (D1). Short-term follow-up with high risk of missing data (D5). |
| **Elbanna (2022)** | S | S | L | L | S | M | NI | **Serious** | Extreme delay (23 yrs) introduces severe selection bias (D2) and missing data bias (D5). No control for confounding (D1). |
| **Fazel (2007)** | S | S | L | L | S | M | NI | **Serious** | Historical series (25-yr interval) with severe selection and missing data bias (D2, D5). No control for confounding (D1). |
| **Liang (2019)** | S | M | L | L | M | M | NI | **Serious** | Retrospective comparative study, but groups not randomized and key confounders not adjusted for (D1). |
| **Han (2023)** | S | M | L | L | M | L | NI | **Serious** | Prospective design helps D5 and D6, but single-arm series has serious risk of bias due to confounding (D1). |
| **Alamo (2006)** | S | M | L | L | S | M | NI | **Serious** | Single-arm, small N, short-term follow-up. Serious risk due to confounding (D1) and missing data (D5). |
| **Sepulveda (2021)** | S | M | L | L | NI | L | NI | **Serious** | Focus on histology/microbiology, but single-arm design with no control for confounding (D1). |
| **Zhao (2024)** | S | M | L | L | M | M | NI | **Serious** | Comparative study but non-randomized with serious confounding bias (D1). Includes revisional cases. |
| **Wang (2022)** | S | M | L | L | S | M | NI | **Serious** | Single-arm, small N, short-term. Serious risk due to confounding (D1) and missing data (D5). |
| **Paiva de Godoy (2019)** | S | M | L | L | S | M | NI | **Serious** | No date of first surgery. Single-arm design with serious confounding bias (D1). |
| **Huang (2024)** | S | M | L | L | S | M | NI | **Serious** | Focus on reoperations only. Large N but single-arm with serious confounding bias (D1). |
| **Raj (2012)** | S | M | L | L | S | M | NI | **Serious** | Prospective series but single-arm with serious confounding bias (D1) and missing data (D5). |
| **Choi (2020)** | S | M | L | L | S | M | NI | **Serious** | Very short-term (0.5y), single-arm. Serious risk due to confounding (D1) and missing data (D5). |
| **Greco (2015)** | S | M | L | L | S | M | NI | **Serious** | No IRB. Long follow-up but single-arm with serious confounding bias (D1). |
| **Vennapusa (2020)** | S | M | L | L | S | M | NI | **Serious** | Focus on predictive factors but single-arm with serious confounding bias (D1). |
| **Lee (2019)** | S | M | L | L | S | M | NI | **Serious** | Long-term (5y) but single-arm with serious confounding bias (D1) and missing data (D5). |
| **Seki (2017)** | S | M | L | L | S | M | NI | **Serious** | 5-year outcomes but single-arm with serious confounding bias (D1) and missing data (D5). |
| **Rezaei (2019)** | S | M | L | L | S | M | NI | **Serious** | Very short-term (0.5y), small N. Single-arm with serious confounding bias (D1). |
| **Cagiltay (2019)** | M | L | L | L | M | L | NI | **Moderate** | **RCT design.** Low risk for confounding (D1). Moderate risk due to potential missing data. |
| **Celik (2015)** | S | M | L | L | S | M | NI | **Serious** | Large N, 2y fu but single-arm with serious confounding bias (D1). |
| **Kota (2012)** | S | M | L | L | S | M | NI | **Serious** | Very short-term fu (0.75y), single-arm with serious confounding bias (D1). |
| **Heap (2008)** | S | M | L | L | S | M | NI | **Serious** | No IRB. Long interval to publication. Single-arm with serious confounding bias (D1). |
| **Wang (2012)** | S | M | L | L | S | M | NI | **Serious** | Comparative study but non-randomized with serious confounding bias (D1). |
| **Lacombe (2011)** | S | M | L | L | S | M | NI | **Serious** | Very small N (9). Single-arm with serious confounding bias (D1). |
| **Velhote (2010)** | S | M | L | L | S | M | NI | **Serious** | Focus on adolescents, small N. Single-arm with serious confounding bias (D1). |
| **Santoro (2003)** | S | M | L | L | S | M | NI | **Serious** | Seminal paper but small initial N, single-arm with serious confounding bias (D1). |
| **Ece (2021)** | S | M | L | L | S | M | NI | **Serious** | IRB approved late. Comparative but non-randomized with serious confounding bias (D1). |
| **Topart (2020)** | S | M | L | L | S | M | NI | **Serious** | Comparative study but non-randomized with serious confounding bias (D1). No IRB. |
| **Yormaz (2018)** | S | M | L | L | S | M | NI | **Serious** | Comparative study but non-randomized with serious confounding bias (D1). No surgery date. |
| **De Paula (2006)** | S | M | L | L | S | M | NI | **Serious** | Pioneering paper. Very short-term fu (0.1y). Single-arm with serious confounding bias (D1). |
| **Ersoz (2015)** | S | M | L | L | S | M | NI | **Serious** | N=1 (case report). No IRB. Single case with serious confounding bias (D1). |
| **Foschi (2024)** | S | M | L | L | S | M | NI | **Serious** | Multicenter, long-term (5y) but low N at 5y fu. Single-arm with serious confounding bias (D1). |
| **Arslan (2018)** | S | M | L | L | S | M | NI | **Serious** | No IRB, no registration. Single-arm with serious confounding bias (D1). |
| **Bilecik (2018)** | S | M | L | L | S | M | NI | **Serious** | Specific population (obese females with T2DM). Single-arm with serious confounding bias (D1). |
| **Caramelli (2018)** | M | L | L | L | M | L | NI | **Moderate** | **RCT design.** Low risk for confounding (D1). Moderate risk due to potential missing data. |
| **Demir (2023)** | S | M | L | L | S | M | NI | **Serious** | No IRB. Multicenter but single-arm with serious confounding bias (D1). |
| **Gulaydin (2022)** | S | M | L | L | S | M | NI | **Serious** | Focuses on technical comparison. Single-arm with serious confounding bias (D1). |
| **Kassir (2018)** | S | M | L | L | S | M | NI | **Serious** | Very small N (7), long-term (5y) fu. Single-arm with serious confounding bias (D1). |
| **Salama (N/A)** | S | M | L | L | S | M | NI | **Serious** | Critical data missing. Single-arm with serious confounding bias (D1). |
| **Santoro (2012)** | S | M | L | L | S | M | NI | **Serious** | Very large N (1020), long-term (5y) but single-arm with serious confounding bias (D1). |
| **Sewefi (2021)** | S | M | L | L | S | M | NI | **Serious** | Large N, 2y fu but single-arm with serious confounding bias (D1). |
| **Sisik (2023)** | S | M | L | L | S | M | NI | **Serious** | Comparative study but non-randomized with serious confounding bias (D1). |
| **Emile (2019)** | S | M | L | L | S | M | NI | **Serious** | Multicenter (10), large N (605) but single-arm with serious confounding bias (D1). |
| **Taskin (2022)** | S | M | L | L | S | M | NI | **Serious** | Very large N (883) but single-arm with serious confounding bias (D1). |
| **Mui (2014)** | S | M | L | L | S | M | NI | **Serious** | N=1 (case report). No IRB. Single case with serious confounding bias (D1). |
| **Lee (2024)** | S | M | L | L | S | M | NI | **Serious** | Comparative study but non-randomized with serious confounding bias (D1). |
| **Zhu (2021)** | S | M | L | L | S | M | NI | **Serious** | Small N (10). No fu duration reported. Single-arm with serious confounding bias (D1). |
| **Melissas (2012)** | S | M | L | L | S | M | NI | **Serious** | 2y fu but single-arm with serious confounding bias (D1). |
| **Melissas (2016)** | S | M | L | L | S | M | NI | **Serious** | Small N (6), registered but single-arm with serious confounding bias (D1). |
| **Shebl (2022)** | S | M | L | L | S | M | NI | **Serious** | Comparative study but non-randomized with serious confounding bias (D1). No surgery date. |
| **Jaworski (2024)** | S | M | L | L | S | M | NI | **Serious** | Multicenter but single-arm with serious confounding bias (D1). No surgery date. |
| **Barski (2022)** | S | M | L | L | S | M | NI | **Serious** | No IRB. Informed consent "day before surgery." Single-arm with serious confounding bias (D1). |
| **Dowgiallo (2023)** | S | M | L | L | S | M | NI | **Serious** | Includes revisional procedures. Single-arm with serious confounding bias (D1). |
| **Alamdari (2023)** | S | M | L | L | S | M | NI | **Serious** | No IRB. Single-arm with serious confounding bias (D1). |
| **Rossoni (2024)** | S | M | L | L | S | M | NI | **Serious** | Long-term (5y) results but single-arm with serious confounding bias (D1). |
| **Balint (2022)** | S | M | L | L | S | M | NI | **Serious** | Small N, registered but single-arm with serious confounding bias (D1). |
| **Luhmann (2018)** | S | M | L | L | S | M | NI | **Serious** | Very long-term follow-up (8y), multicenter but single-arm with serious confounding bias (D1). |

**Legends:**

ROBINS-I Domains:
D1: Bias due to confounding
D2: Bias in selection of participants
D3: Bias in classification of interventions
D4: Bias due to deviations from intended interventions
D5: Bias due to missing data
D6: Bias in measurement of outcomes
D7: Bias in selection of the reported result

Risk of Bias Judgments:
L: Low
M: Moderate
S: Serious
NI: No Information
NA: Not Applicable

**References**

1. Page MJ, McKenzie JE, Bossuyt PM, et al. The PRISMA 2020 statement: an updated guideline for reporting systematic reviews. BMJ. 2021;372:n71. doi:10.1136/bmj.n71
2. Wells GA, Shea B, O'Connell D, et al. The Newcastle-Ottawa Scale (NOS) for assessing the quality of nonrandomised studies in meta-analyses. 2014. Available from: <http://www.ohri.ca/programs/clinical_epidemiology/oxford.asp>
3. Moola S, Munn Z, Tufanaru C, et al. Chapter 7: Systematic reviews of etiology and risk. In: Aromataris E, Munn Z (Editors). JBI Manual for Evidence Synthesis. JBI; 2020. Available from: [https://synthesismanual.jbi.global](https://synthesismanual.jbi.global/)
4. Sterne JA, Hernán MA, Reeves BC, et al. ROBINS-I: a tool for assessing risk of bias in non-randomised studies of interventions. BMJ. 2016;355:i4919. doi:10.1136/bmj.i4919
